# Immunohistochemical overexpression of BCL-2 protein predicts an inferior survival in patients with primary central nervous system diffuse large B-cell lymphoma

**DOI:** 10.1097/MD.0000000000017827

**Published:** 2019-11-11

**Authors:** Yi Chen, Hong Chen, Lushan Chen, Xiaoyun Zheng, Xiaozhu Yang, Zhihong Zheng, Jing Zheng, Ting Yang, Tingbo Liu, Yinghong Yang, Jianda Hu

**Affiliations:** Fujian Provincial Key Laboratory on Hematology, Fujian Medical University Union Hospital, Fujian Institute of Hematology, Fuzhou, Fujian, China.

**Keywords:** BCL-2, clinical features, c-MYC, diffuse large B-cell lymphoma, primary central nervous system, prognosis

## Abstract

This study was designed to analyze the clinical characteristics and prognostic value of c-MYC and BCL-2 proteins expression in patients with primary central nervous system diffuse large B-cell lymphoma (PCNS-DLBCL).

82 patients newly diagnosed with PCNS-DLBCL, from January 2008 to November 2018, were enrolled in this study. Clinical characteristics, immunohistochemical features, laboratory examinations, and treatment outcome were analyzed among these patients.

Among these 82 cases, 45 were males (54.9%) and 37 were females (45.1%). Age ranged from 16 to 78 years old, and 29 patients (35.4%) were elder than 60 years old, with median age at 57 years old. According to Hans classification, 25 were accounted for origin of germinal center B-cell (GCB) subtype (30.5%) and 49 were accounted for non-GCB subtype (59.8%), respectively. Eight patients were unclassified due to lack of detailed pathological results. The median survival of these 82 patients was 30 months, and 1-year, 3-year, and 5-year overall survival (OS) rate was 59.7%, 44.6%, and 34.1%, respectively. Patients treated with sequential HD-MTX based chemotherapies showed a superior prognosis than those without. In combination with rituximab, the outcome was further improved. The median OS was 55 months in HD-MTX + R group, 27 months in HD-MTX group, and 9 months in other groups, respectively. Univariate analysis identified age ≥60, ECOG score ≥ 2 points, and overexpression of BCL-2 protein (≥85%) were adverse prognostic factors for OS. Co-expression of c-MYC (≥40%) and BCL-2 (≥50%) proteins was associated with poor ECOG score, high Ki-67 expression, and trended towards an inferior outcome. Gender, lesion location, number of lesions, lactic dehydrogenase (LDH), cell of origin, BCL-6 protein expression, expression of c-MYC protein alone and Ki-67 ≥85% had no significant impact on OS.

In patients with PCNS-DLBCL, age ≥60 years old, ECOG score ≥2 points, and overexpression of BCL-2 protein (≥85%) were associated with a poor survival. HD-MTX based chemotherapies in combination with rituximab could improve the prognosis.

## 1 Introduction

Primary central nervous system lymphoma (PCNSL) is a rare form of extranodal non-Hodgkin lymphoma (NHL) limited to the brain, spinal cord, leptomeninges or eyes, and accounts for approximately 2% of all primary central nervous system tumors, with an incidence of 0.44 per 100,000.^[1]^ Approximately 90% of PCNSL cases are diffuse large B-cell lymphoma (DLBCL), with rather distinct pathophysiology and prognostic features in comparison to systemic DLBCL.^[2]^ The majority of PCNS-DLBCL closely resemble a post-germinal center or an activated B-cell (ABC) immunophenotype (CD10-, BCL-6+, MUM1/IRF4+).^[3]^

Treatment of PCNS-DLBCL has evolved during the past few decades but remains poor, and no uniform consensus on the optimal treatment regimen exists. High-dose methotrexate (HD-MTX) plays a crucial role in PCNS-DLBCL and is considered the backbone of multimodal therapy, which can include other chemotherapeutic agents. Additionally, prognostic factors of PCNS-DLBCL were not fully recognized. International Extranodal Lymphoma Study Group (IELSG) revealed old age and poor clinical performance status (PS) have the strongest negative impact on prognosis. Besides these, increased lactic dehydrogenase (LDH) levels, elevated cerebrospinal fluid (CSF) protein levels, and involvement of deeper brain regions might also associate with a poorer prognosis.^[4–6]^

With the cell of origin (COO) concept, DLBCL has been divided into germinal center B-cell (GCB)-like subtype and ABC-like subtype according to the “gold standard” method based on gene expression profiling (GEP) of RNA from fresh frozen tissue using microarray technology. Because of the high prices and strict requirements regarding tissue, routine use of this method is difficult.^[7]^ As a result, some immunohistochemistry-based algorithms have been described, including the Hans model. Recently, there is growing interest in the double expressor lymphomas (DELs), defined as co-expression of 2 oncogenes (MYC and BCL2) based on immunohistochemical staining. Previous studies found that the prognostic effects of COO were lost when adjusting for DEL in patients with systematic DLBCL, which suggested that MYC/BCL2 co-expression by immunohistochemical analysis is a more reliable predictor of prognosis than COO in patients with systematic DLBCL, but there was still undefined in PCNS-DLBCL.^[8]^

Thus, we retrospectively analyzed the clinical features and prognosis of 82 patients with PCNS-DLBCL in our center, and explored the prognostic value of expression of c-MYC and BCL-2 proteins in PCNS-DLBCL patients.

## 2 Patients and methods

### 2.1 Patients characteristics and treatment protocols

Between March 2008 and October 2018, 82 patients newly diagnosed with PCNS-DLBCL, were enrolled in this retrospective analysis. The pathological diagnosis of PCNS-DLBCL was made according to 2008 revision of the World Health Organization (WHO) classification of lymphoid neoplasms. Patients with extraneural involvement or immune deficiency were excluded in the study. The following clinicopathological information was collected, including patients’ age, gender, Eastern Cooperative Oncology Group (ECOG) score, lesion location, number of lesions, LDH, and treatment protocols. This study was approved by local Ethics Committee.

Treatment protocol for PCNS-DLBCL evolved over the past decade in our institution, and the standard first line treatment has not been established. At first, patients were treated with CHOP or CHOP-like regimen with or without rituximab as patients with systematic DLBCL, but the outcome was unsatisfactory. Subsequently, HD-MTX was performed for this subtype in combination with other chemotherapeutic agents and/or WBRT, and the prognosis was significantly improved. In particular, combination of rituximab to HD-MTX demonstrated significantly improved response and survival rate compare to single agent therapy. However, there is no consensus on the optimal dose of HD-X or on the role of radiation in combination with methotrexate in the management of PCNS-DLBCL. Currently, HD-MTX-based chemotherapy in combination with rituximab and/or other chemotherapeutic agents were recommended in our center.

### 2.2 Immunohistochemical staining and interpretation

Tissues were fixed in 10% formalin and embedded in paraffin. Sections were stained with hematoxylin and eosin and immunohistochemical analysis was performed. The following antibodies were used: CD10, BCL-6, MUM1, c-MYC, and BCL-2. Formalin-fixed and paraffin-embedded tissues slides were performed according to the protocols for automated immunohistochemistry using the Ventana Discovery XT automatic platform (Ventana Medical Systems, Tucson, AZ). The cut-off for positivity of CD10, BCL-6, and MUM1 was 30% or higher for each, the cutoff was 40% or higher for c-MYC, and 50% or higher for BCL-2.^[9]^ The staining pattern for c-MYC protein was distinctly nuclear, whereas the staining for BCL-2 protein showed a well-defined cytoplasmic staining pattern. Cell of GCB or non-GCB origin was determined by Hans classification.^[10]^

### 2.3 Statistical analysis

Overall survival (OS) was defined as the time from the initial diagnosis to death or the last follow-up. Patients who were alive at the last follow-up were treated as censored. The difference between patient subgroups was analyzed by the Chi-squared test for categorical variables and by the t-test for continuous variables. Survival data were analyzed by means of the log-rank test, and survival curves were made using the Kaplan-Meier method. All of the data were analyzed with SPSS 21.0 software (IBM Corp., Armonk, NY). In all above statistical analysis, *P* value less than .05 was considered statistically significant.

## 3 Results

### 3.1 Clinical and immunohistochemical features of PCNS-DLBCL

Among these 82 patients, 45 cases were male, and 37 cases were female. Age ranged from 16 to 78 years old, and 29 patients (35.4%) were elder than 60 years old, with median age at 57 years old. 34 patients (41.5%) presented with deep region involvement, while another 48 patients (58.5%) were not. Elevated LDH was only found in 9 patients (11.8%). 57 patients (69.5%) presented with a single lesion, and 25 patients (30.5%) had multiple lesions. The detailed clinical features of these 82 patients with PCNS-DLBCL were listed at Table 1.

**Table 1:**
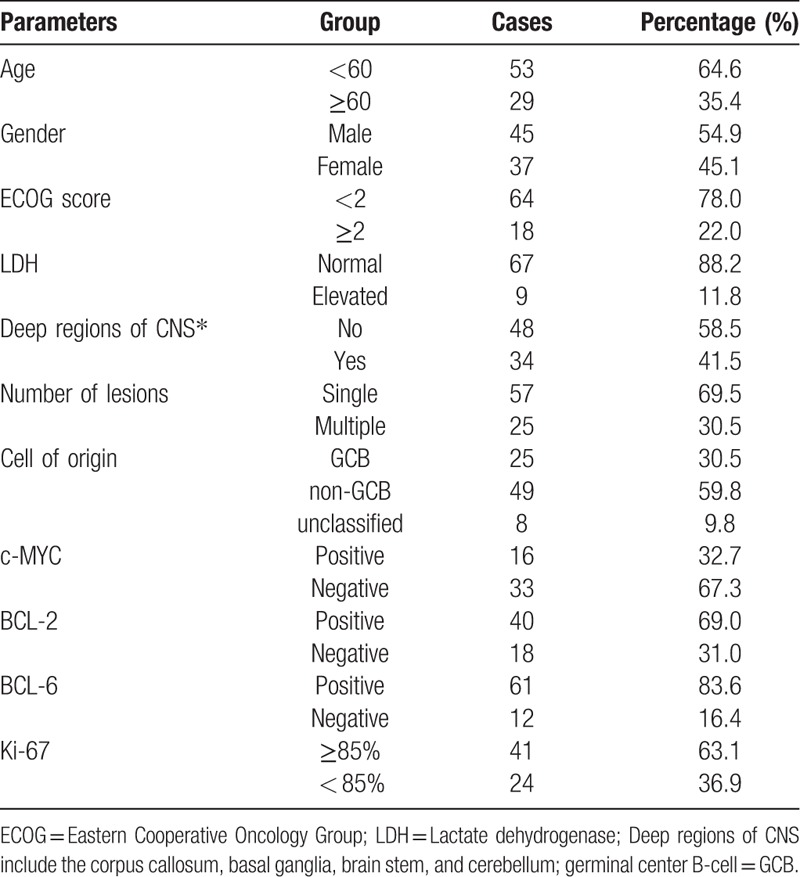
Clinical and immunohistochemical features.

According to Hans classification, 25 were accounted for origin of germinal center B-cell (GCB) subtype (30.5%) and 49 were accounted for non-GCB subtype (59.8%), respectively, while 8 patients was unclassified due to lack of immunohistochemical detail. The 32.7% of cases (16/49) were positive for c-MYC and 69.0% of cases (40/58) were positive for BCL-2 protein, respectively. 22.4% of patients (11/49) were positive for both c-MYC and BCL-2 proteins, and was considered as double-expression lymphoma (DEL).

Various clinicopathologic characteristics were compared between PCNS-DLBCL patients with GCB subtype and non-GCB subtype. We also compared the same clinical features between patients with and without DEL (Table 2). Remarkably, more frequent DEL was observed in non-GCB subtype in comparison to GCB subtype (33% vs 5%, *P* = .023). Additionally, DEL was associated with poorer ECOG score and higher Ki-67 expression.

**Table 2:**
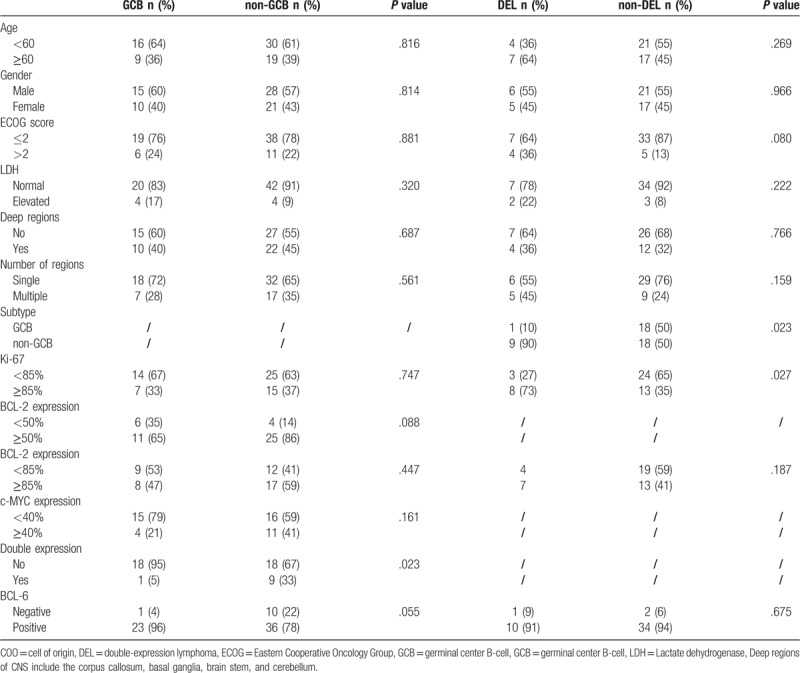
Patients features based on COO concept and MYC/BCL2 co-expression.

### 3.2 Treatment protocols

In this analysis, after initial surgical resection, 66 patients were treated with chemotherapies alone or in combination with radiotherapy, while the other 16 patients refused to receive further therapies. In the chemotherapy group, 55 patients were treated with HD-MTX based chemotherapies, including 31 patients in combination with rituximab, and 11 patients were treated with other chemotherapy agents or radiotherapy. Dosage of MTX ranged from 1 to 5 g/m^2^, which was determined by patients’ condition. Dexamethasone was frequently used in chemotherapies group. Other therapeutic agents included cytarabine, cyclophosphamide, doxorubicin, vincristine and temozolomide. 10 patients received subsequent radiotherapy in combination with chemotherapies. Only one patient received autologous stem cell transplantation (ASCT).

### 3.3 Treatment outcome and prognostic factors

**Figure 1:**
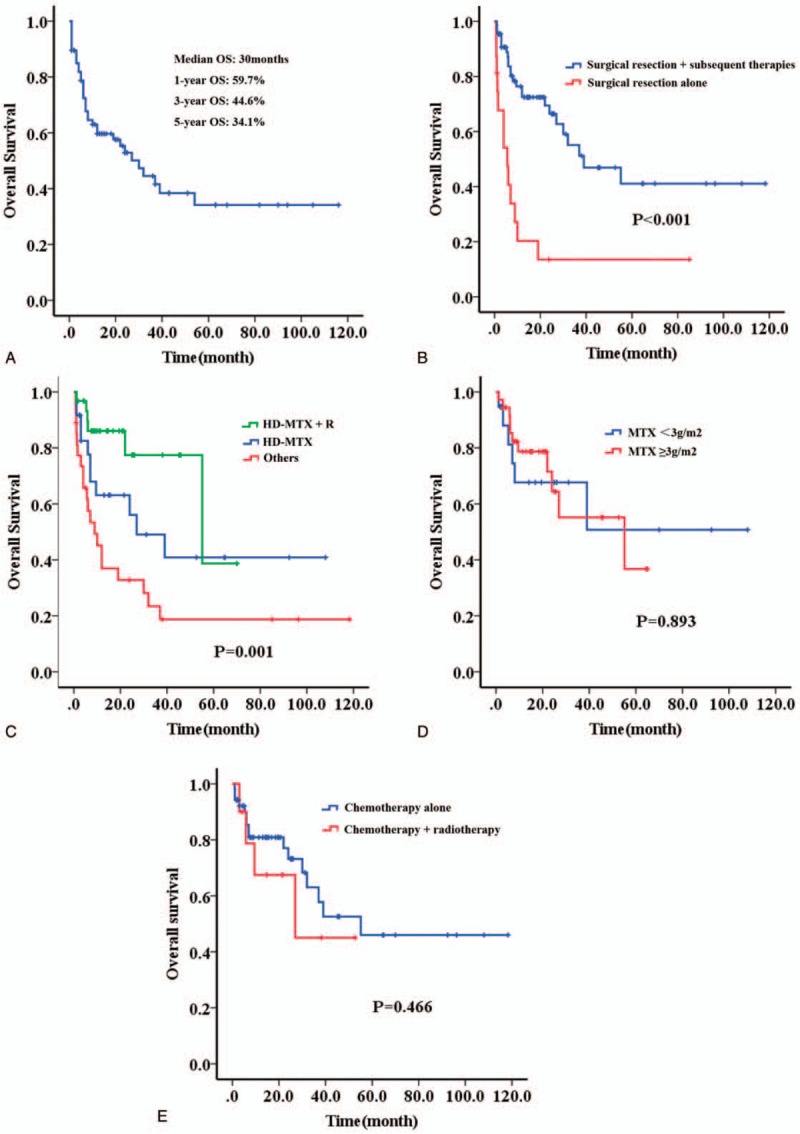
Survival curve of patients with PCNS-DLBCL in the study. (A) All of these 82 patients; (B) patient treated with surgical section alone or in combination with subsequent chemotherapy and radiotherapy; (C) patients treated with HD-MTX based chemotherapies, or HD-MTX based chemotherapies + R, or other agents; (D) patients treated with different dosage of MTX; (E) patients treated with chemotherapy alone or in combination with radiotherapy.

The follow-up time ranged from 1 to 118 months, and median follow-up time was 12 months. For the all enrolled patients, the median OS was 30 months in our data, and 1-year, 3-year, and 5-year overall survival (OS) rate was 59.7%, 44.6%, and 34.1%, respectively (Fig. 1A). It was noticed that subsequent chemotherapy and radiotherapy after surgical resection could improve the prognosis in comparison to surgical resection alone (Fig. 1B). Importantly, patients treated with sequential HD-MTX based chemotherapies showed a superior prognosis than those without, and the outcome was further improved when it in combination with rituximab (R), as it was shown in Figure 1C. The median OS was 55 months in high-dose HD-MTX + R group, 27 months in HD-MTX group, and 9 months in other groups, respectively (*P* = .001). No significant difference of OS was observed between dosage of MTX ≥3 g/m^2^ group and MTX <3 g/m^2^ group (Fig. 1D). Additionally, combination with sequential radiotherapy did not improve the outcome of PCNS-DLBCL, compared to chemotherapy alone (*P* = .574, Fig. 1E).

**Figure 2:**
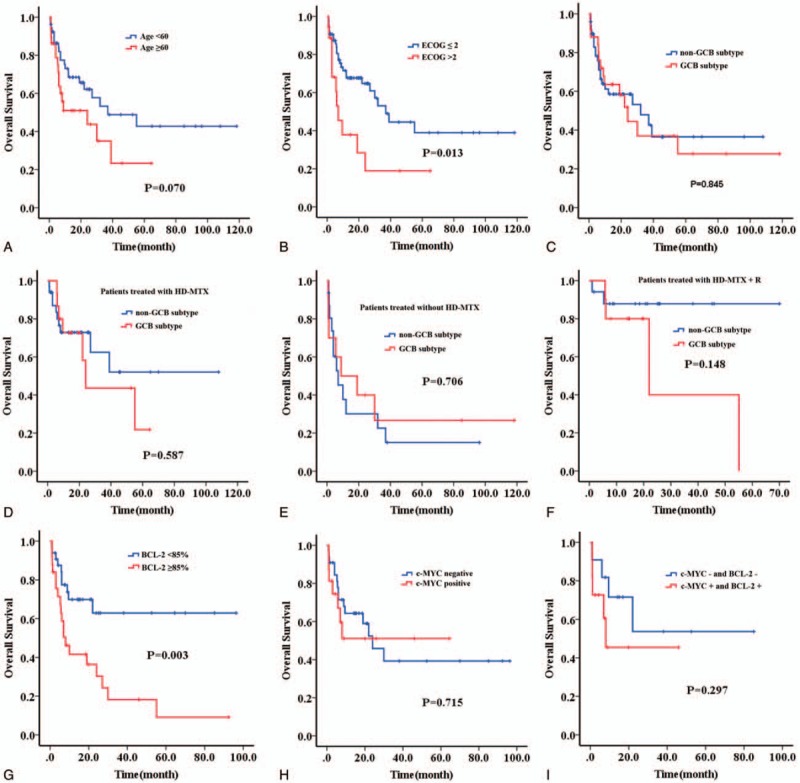
Survival curve of patients with different clinicopathologic parameters. (A) Age; (B) ECOG score; (C) cell of origin in all patients; (D) cell of origin in patients treated with HD-MTX based chemotherapies; (E) cell of origin in patients treated without HD-MTX based chemotherapies; (F) cell of origin in patients treated with HD-MTX based chemotherapies and rituximab; (G) BCL-2 protein; (H) c-MYC protein; (I) c-MYC and BCL-2 proteins.

In order to identify the potential prognostic factors of PCNS-DLBCL, we performed a univariate analysis for OS. As it was listed at Table 3, we identified that age ≥60 and ECOG score ≥ 2 points were adverse clinical prognostic factors for OS (Fig. 2A-B). No significantly prognostic value was found in the following clinicopathologic parameters, including gender, lesion location, number of lesions, LDH, cell of origin, BCL-6 protein expression, and Ki-67 ≥85%. None independent prognostic factor was noticed in multivariable survival analysis.

**Table 3:**
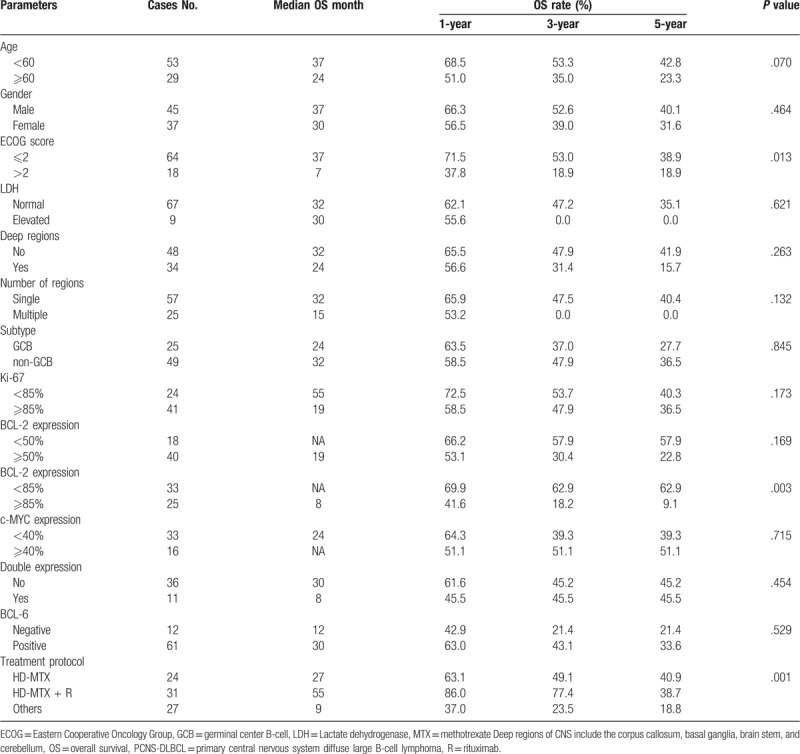
Prognostic factors of PCNS-DLBCL.

### 3.4 Prognostic value of COO concept and MYC/BCL2 protein expression

As described, the majority of the cases were classified as non-GCB subtype based on the immunohistochemical data. No significant difference of prognosis was observed between the GCB subtype and non-GCB cases among the whole series, but patients with non-GCB subtype trended towards a superior response in HD-MTX group and HD-MTX + R group (Fig. 2C–F). Additionally, it should be emphasized that overexpression of BCL-2 protein (≥85%) was a significant adverse prognostic factor for OS, while expression of c-MYC protein alone had no impact on OS (Fig. 2G-H). In our data, patients with DEL trended towards an inferior prognosis compared to those without (Fig. 2I).

## 4 Discussion

PCNS-DLBCL is a rare entity of DLBCL with an inferior responses and prognosis to current treatment regimens, and the optimal protocol for this subtype has not been fully established. Corticosteroids lead to decreased tumor-associated edema and may result in partial radiographic regression due to their lymphotoxic effects. After an initial response to corticosteroids, most patients quickly have relapsing disease.^[11]^ Currently, surgical resection is not considered the standard treatment in PCNS-DLBCL given the multifocal nature of this tumor.^[12]^ In our data, patients treated with surgical section alone shown significantly inferior outcome, compared to those treated with sequential chemotherapies or radiotherapy. Historically, radiotherapy was performed as first-line therapy for PCNS-DLBCL, resulting in a high proportion of radiographic responses, but with early relapse and increased neurotoxicity.^[13–15]^ Thus, radiotherapy was no longer a routinely recommended treatment for patients with newly diagnosed PCNS-DLBCL, and our results proved that it could not further improve the prognosis based on subsequent chemotherapies after surgical section.

As it was reported by previous literatures, HD-MTX in combination with other chemotherapeutic agents seems the most effective regimen for newly diagnosed PCNS-DLBCL.^[16–17]^ However, there is no consensus on the optimal dose of MTX in the management of newly diagnosed PCNS-DLBL. Doses of MTX of at least 3 g/m^2^ achieve sufficient therapeutic concentrations in the brain parenchyma and CSF, which might lead to more durable treatment responses.^[18]^ In our analysis, dosage of MTX ranged from 1 to 5 g/m^2^, and the outcome of patients treated with HD-MTX was significantly superior than those without HD-MTX. However, no significant difference of OS was observed between dosage of MTX ≥3 g/m^2^ group and MTX <3 g/m^2^ group in our data, but we could not further compare the difference of each dosage due to the limited cases. Thus, a prospective randomized clinical trail was still needed for exploring the best dosage of MTX in treatment of PCNS-DLBCL. Rituximab is a chimeric monoclonal antibody targeting the CD20 antigen. Some former studies revealed that CR rates were higher in induction regimens that include rituximab compared with chemotherapy regimens without rituximab.^[19,20]^ In our data, we could not calculate the CR rate exactly because of ununiform efficacy evaluation standard, but improvement of OS was confirmed when HD-MTX in combination with rituximab, compared to HD-MTX alone. Our analysis displayed that patients with non-GCB subtype seemed more sensitive to HD-MTX and rituximab, but we could not analyze this difference between two subtypes in the comparable baseline because of only few cases. Thus, it needs to be further confirmed in prospective clinical trials with large samples.

Some former studies indicated that expression of c-MYC protein was frequently observed and was associated with poor outcome in PCNS-DLBCL in comparison to systemic DLBCL.^[21,22]^ The proportion of c-MYC expression ranged from 30% to 90% of cases reported by previous literature.^[23,24]^ In our study, 32.7% of cases presented with c-MYC expression, which was similar to systematic DLBCL. Additionally, our data revealed that expression of c-MYC protein alone had no impact on OS. Besides, the prognostic value of BCL-2 expression in PCNS-DLBCL was still unclear up to now. In Tapia's study, BCL-2 positivity was observed in 71% of PCNS-DLBCL cases, but it had no relationship with outcome.^[24]^ In contrast, Kim and Makino studies demonstrated that BCL-2 overexpression had a significantly poorer OS in patients with PCNS-DLBCL.^[25,26]^ BCL-2 expression was detected in 69.0% of cases in our series, which was within the range described previously. It should be noticed that BCL-2 expression (≥50%) had no significant influence on prognosis, but it was a strongly adverse prognostic factor when it was overexpression (≥85%).

MYC/BCL-2 double-hit lymphoma (DHL) was associated with resistance or a poor response to therapy and an inferior survival rate in systemic DLBCL.^[27–28]^ MYC/BCL-2 DEL assessed by immunohistochemistry has also been shown to have an adverse prognostic effect on systemic DLBCL, independent of COO subtype.^[29–30]^ In systematic DLBCL, most DHL have a GCB-like origin, whereas the DEL have an ABC-like origin.^[31]^ Our data exhibited that DEL was predominantly observed in non-GCB subtype, and that was similar with systemic DLBCL. Furthermore, DEL are always associated with poor ECOG, advanced diseases, a higher proliferation index, and a higher International Prognostic Index (IPI) in systemic DLBCL.^[32,33]^ Our study had shown that patients with DEL more frequently presented with poor ECOG and higher Ki-67 in comparison to those without.

Currently, the prognostic value of c-MYC/BCL-2 co-expression in PCNS-DLBCL does not allow a unified conclusion to be drawn. In Gill study, no prognostic significance of MYC and/or BCL-2 expression was found in survival.^[22]^ In contrast, Guo et al found that MYC expression alone and MYC/BCL-2 co-expression was significantly associated with poor outcome in patients with PCNS-DLBCL.^[34]^ Kim et al summarized that patients with MYC/BCL-2 co-expression exhibited worse progression-free survival (PFS) in the MTX-treated group, and the same conclusion was got in Shi study.^[25,23]^ Similarly, our research found that patients with c-MYC/BCL2 co-expression trended towards a poor prognosis. However, due to limited cases of this study, we could not compare the survival of PCNS-DLBCL patients with and without DEL under the treatment of HD-MTX alone or in combination with rituximab. Thus, prognostic value of c-MYC/BCL2 co-expression in PCNS-DLBCL still needs to be further studied based on the treatment of HD-MTX and rituximab.

Protein phosphorylation and de-phosphorylation play critical roles as a mode of signal transfer in biological processes. Protein phosphorylation is estimated to affect 30% of the proteome and is a major regulatory mechanism that controls many basic cellular processes. Furthermore, integration of the phosphorylation results with protein–protein interaction and transcription factor binding data revealed novel regulatory modules. Previous studies had demonstrated the important role played by protein phosphorylation in lymphoma development, and it needed to be studied further in PCNS-DLBCL.^[35–37]^

There were still some limitations that must be considered when interpreting these results. Because of retrospective design and long period of observation, some information bias could not be avoided. Patients’ treatment regimen was partly affected by their condition, and the dosage and cycle of one drug was no uniform. Recently, some novel agents, such as lenalidomide, ibrutinib, PD-1 inhibitor, and chimeric antigen receptor (CAR)-T-cell therapy are increasingly used in the treatment of PCNS-DLBCL.^[38–41]^ Some previous studies had shown promising results, but they were still in clinical trials and needed to be further studied.

In conclusion, treatment for PCNS-DLBCL has advanced significantly with improved survival for this rare and aggressive lymphoma, but the long-term survival remains poor. Older age, poor ECOG score, overexpression of BCL-2 (≥85%) were significant adverse prognostic factor for OS. The optimal treatment approach has not been well established, and HD-MTX based chemotherapies in combination with rituximab and/or other chemotherapeutic agents were effective regimens for newly diagnosed PCNS-DLBCL.

## Acknowledgments

The authors would like to express their gratitude to all who helped them during this study.

## Author contributions

**Data curation:** Hong Chen.

**Formal analysis:** Yi Chen, Hong Chen, Lushan Chen.

**Investigation:** Lushan Chen, Xiaoyun Zheng.

**Methodology:** Lushan Chen, Jing Zheng, Ting Yang, Tingbo Liu.

**Project administration:** Yi Chen, Hong Chen, Tingbo Liu, Yinghong Yang, Jianda Hu.

**Resources:** Xiaoyun Zheng, Xiaozhu Yang, Zhihong Zheng, Yinghong Yang.

**Software:** Yi Chen.

**Validation:** Xiaozhu Yang.

**Visualization:** Zhihong Zheng.

**Writing – original draft:** Yi Chen.

**Writing – review & editing:** Jianda Hu.
